# Efficacy of three chemical disinfectants and steam against *Clostridioides difficile* endospores on nylon carpet with two different backing systems

**DOI:** 10.1128/aem.00861-25

**Published:** 2025-06-20

**Authors:** Jinge Huang, Angela Fraser, Xiuping Jiang

**Affiliations:** 1 Department of Food, Nutrition, and Packaging Sciences, Clemson University 2545, Clemson, South Carolina, USA; Centers for Disease Control and Prevention, Atlanta, Georgia, USA

**Keywords:** *Clostridioides difficile *endospores, disinfection efficacy, hydrogen peroxide, steam, carpet backing

## Abstract

Carpet could serve as a potential vehicle for transmitting *Clostridioides difficile* endospores, a leading cause of healthcare-associated infections. To develop carpet disinfection practices, a validated recovery method and standard efficacy testing method are necessary. The recovery method was optimized to detect *C. difficil*e endospores on carpet by using different concentrations of Tween-80 and varying stomaching times. Adding 0.2% Tween-80 followed by stomaching for 3 min and sonication increased the recovery rate of *C. difficile* endospores to >60%. The efficacy of three disinfectants (two hydrogen peroxide-based products, A and B, and one chlorine-based product, C) and steam was tested against *C. difficile* endospores on two types of nylon carpet (with water-permeable backing and waterproof backing). The results showed that product B was the most effective among the chemical disinfectants, achieving a 5.8 and 4.9 log_10_ CFU reduction of *C. difficile* endospores in 30 min on carpets with water-permeable and waterproof backings, respectively. A steam treatment of 120 s resulted in 4.9 and >6.0 log_10_ CFU reduction on water-permeable and waterproof backings, respectively. On water-permeable backing, 30 s steam treatment followed by application of H_2_O_2_-based products A and B resulted in a 4.4 and >6.1 log_10_ CFU reduction of *C. difficile* endospores, respectively. Overall, a 120 s steam treatment was more effective than any of the three disinfectants tested. Disinfectant efficacy varied by carpet backing type, underscoring the need for guidance in carpet selection for healthcare safety.

**IMPORTANCE:**

*Clostridioides difficile*, a spore-forming anaerobic bacterium and a leading cause of healthcare-associated infections, can be transmitted from the floor to other surfaces via air movement. Therefore, disinfection of all floors after cleaning, regardless of type, might be necessary to prevent recurrent *C. difficile* infections among patients. To develop carpet disinfection practices, a validated recovery method and standard efficacy testing method are necessary. In this study, we first optimized the spore recovery method from carpets. Next, our study demonstrated that carpet backing affected the efficacy of chemical disinfectants and steam against *C. difficile* endospores. Steam was particularly effective on carpets with waterproof backing, while only product B showed strong efficacy on carpets with water-permeable backing. When steam was combined with a chemical disinfectant, the efficacy of both H_2_O_2_-based products against *C. difficile* endospores was enhanced. These findings can inform the development of carpet disinfection practices.

## INTRODUCTION

*Clostridioides difficile* is a gram-positive, spore-forming anaerobic bacterium and a leading cause of healthcare-associated infections (1). Patients with a *Clostridioides difficil*e infection (CDI) have a significantly higher mortality rate (40.9%) compared to those without a CDI (7.4%) (2). Many CDIs are transmitted via contact with contaminated surfaces, necessitating an antimicrobial treatment. While high-contact surfaces are routinely disinfected to prevent pathogen transmission, floors are often considered low risk (3, 4). However, some reports suggest floors, especially carpeted flooring, could act as a vehicle for *C. difficile* endospore spread in healthcare settings (5, 6). Recent epidemiological evidence suggests pathogens can move from the floor to other surfaces via air movement (7, 8). Therefore, disinfection of all floors after cleaning, regardless of type, might be necessary to prevent recurrent CDIs among patients.

Floors in healthcare facilities are typically constructed of non-porous materials like vinyl tile (9). However, in long-term care facilities, both non-porous and porous floorings, such as nylon carpet, are used. Porous surfaces like carpet are challenging to treat due to their complex structure and ability to absorb dust, microbes, and moisture, allowing pathogens to persist in the fibers (10). Vacuuming, while effective in removing dust and moisture, can facilitate the spread of pathogens by resuspending them into the air (11, 12). Bacterial spores, because of their small size and lightweight nature, are more likely to be resuspended from carpet compared to non-porous surfaces (11). This increased potential for airborne transmission highlights the importance of carpet disinfection protocols. As carpets are non-launderable, in-place disinfection methods are required. These points underscore the need for validated disinfection protocols for use on carpeted floors to prevent the transmission of pathogens like *C. difficile* in healthcare settings.

The U.S. Environmental Protection Agency (EPA) maintains List K: antimicrobial products registered with EPA for claims against *C. difficile* spores (13). According to this list, two major types of antimicrobials have known efficacy against *C. difficile* endospores: chlorine- and hydrogen peroxide-based disinfectants (13). The use of chlorine-based products at high concentrations or over extended periods can result in surface damage (14). Hydrogen peroxide-based disinfectant sprays, reported to be efficacious against human norovirus and *C. difficile* endospores on non-porous and porous surfaces, could be a potential alternative to chlorine-based disinfectants (15). Moreover, H_2_O_2_-based disinfectants do not visibly damage fabrics, whereas chlorine-based disinfectants do, such as bleaching fabrics (16, 17). In addition to chemical disinfectants, physical methods, such as steam, can be sporicidal. While EPA does not regulate the efficacy of steam-vapor generation devices, our previous study showed steam could rapidly inactivate human norovirus surrogates on carpet without visible damage (18). Additionally, no research investigating the application of steam against *C. difficile* endospores on carpet has been published, and a combination of steam and chemical disinfectants against *C. difficile* endospores has not been explored either.

Fiber type was a common research focus on multiple microbial persistence and disinfection studies on fabrics (18–22). Nylon is the most popular material used to construct carpet in healthcare facilities due to its durability, cost-effectiveness, and functionality (23, 24). Carpet backing, an essential component in its construction, is typically constructed of a fiber-binding primary backing and a functional secondary backing with waterproof materials, such as polyvinyl chloride or polyurethane (25). Though the secondary backing is constructed of waterproof materials, grids are usually structured to give the backing flexibility. Some studies revealed the role of fibers in attracting dust and affecting disinfectant efficacy (18, 26, 27), but the disinfection efficacy of water-permeable backing (WPerB) and waterproof backing (WProB) of carpet has not been compared (28).

Despite the demonstrated potential of hydrogen peroxide-based products and steam for inactivating *C. difficile* endospores on stainless steel (15), a critical gap remains in both efficacy testing methodology and effective disinfection strategies for soft surfaces such as carpet, particularly given that current disinfection practices are largely ineffective against *C. difficile* endospore contamination. To address this, our study first optimized a method for recovering *C. difficile* endospores from carpet, enabling reliable evaluation of disinfection efficacy on soft surfaces. We then conducted disinfection efficacy studies on carpet surfaces with two types of carpet backing systems to assess and improve upon existing disinfection limitations.

## RESULTS

### Recovery optimization of *C. difficile* endospores from nylon carpet

Both Tween-80 concentration and stomaching time were optimized to recover *C. difficile* endospores from nylon carpet with WPerB in the presence and absence of soil. After ultrasonication, the recovery rates of *C. difficile* spores with 0.02%, 0.1%, and 0.2% Tween-80 were 11.3%, 15.7%, and 20.8% without soil and 10.9%, 14.7%, and 18.9% with soil (equivalent to 5% fetal bovine serum), respectively (Table 1). Because recovery rates for 0.2% Tween-80 were significantly (*P* < 0.05) higher than 0.02% Tween-80 in the presence or absence of soil load, 0.2% was used for the following sections in this study. By incorporating a stomaching step, recovery rates with sonication followed by stomaching for 1, 2, and 3 min at 200 rpm were increased to 59.8%, 72.1%, and 89.4% in the absence of soil, respectively, and 45.3%, 64.2%, and 66.5% in the presence of soil, respectively (Table 2). A longer stomaching time (3 min) significantly (*P* < 0.05) improved recovery rate compared to 1 min stomaching.

**TABLE 1:** Effect of Tween-80 concentration on *C. difficile* endospore recovery rate from WPerB carpet

Concentration of Tween-80 (%)	Recovery rate (%)^*a*^	
	Without soil load	With soil load
0.02	11.3 ± 1.8^A^	10.9 ± 1.1^A^
0.1	15.7 ± 4.1^AB^	14.7 ± 2.0^AB^
0.2	20.8 ± 1.4^B^	18.9 ± 2.8^B^

^*a*^ The data are expressed as means ± standard deviations from duplicates in each of two independent experiments. The recovery rate was calculated based on plate count data (CFU). Values with different letters within the same column indicate a significant difference (*P* < 0.05).

**TABLE 2:** Effect of stomaching time on *C. difficile* endospore recovery rate from WPerB carpet

Stomaching time (min)	Recovery rate (%)^*a*^	
	Without soil load	With soil load
1	59.8 ± 3.3^A^	45.3 ± 7.3^A^
2	72.1 ± 6.4^AB^	64.2 ± 5.9^B^
3	89.4 ± 13.0^B^	66.5 ± 7.4^B^

^*a*^ The data are expressed as means ± standard deviations from duplicates in each of two independent experiments. The recovery rate was calculated based on plate count data (CFU). Values with different letters within the same column indicate a significant difference (*P* < 0.05).

**Fig 1:**
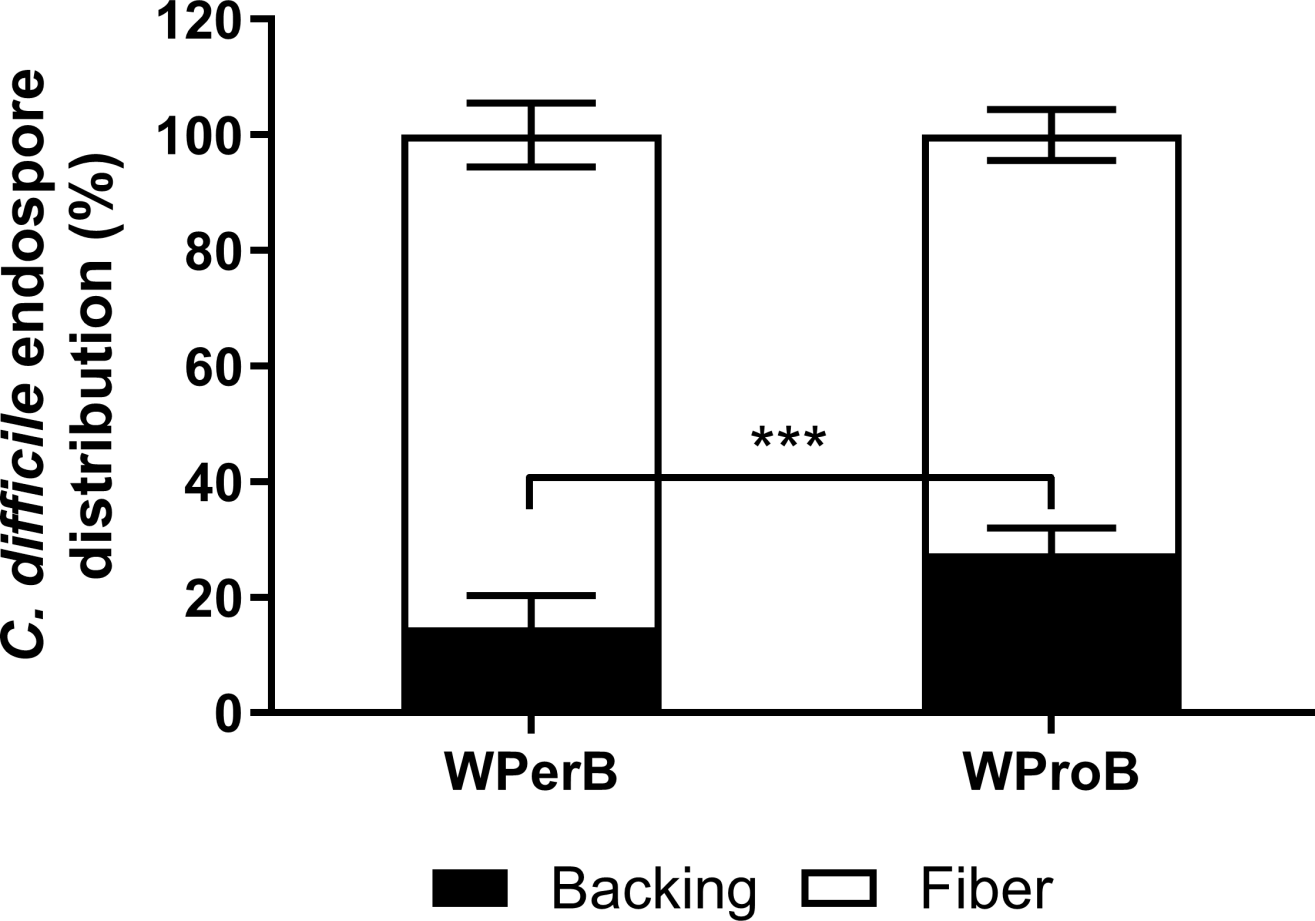
Distribution of *C. difficile* endospores on WPerB and WProB carpets. The *P* value between the two types of carpet was ≤0.001 (***).

To further analyze the distribution of *C. difficile* endospores in the carpet coupon, we enumerated the spore population in both fibers and backings separately. Not all *C. difficile* endospores were in the fibers of carpet, as 14.75% and 27.57% of *C. difficile* endospores were absorbed by the backing of WPerB and WProB, respectively (Fig. 1).

### Efficacy of chemical disinfectants and steam against *C. difficile* endospores on carpet

**Fig 2:**
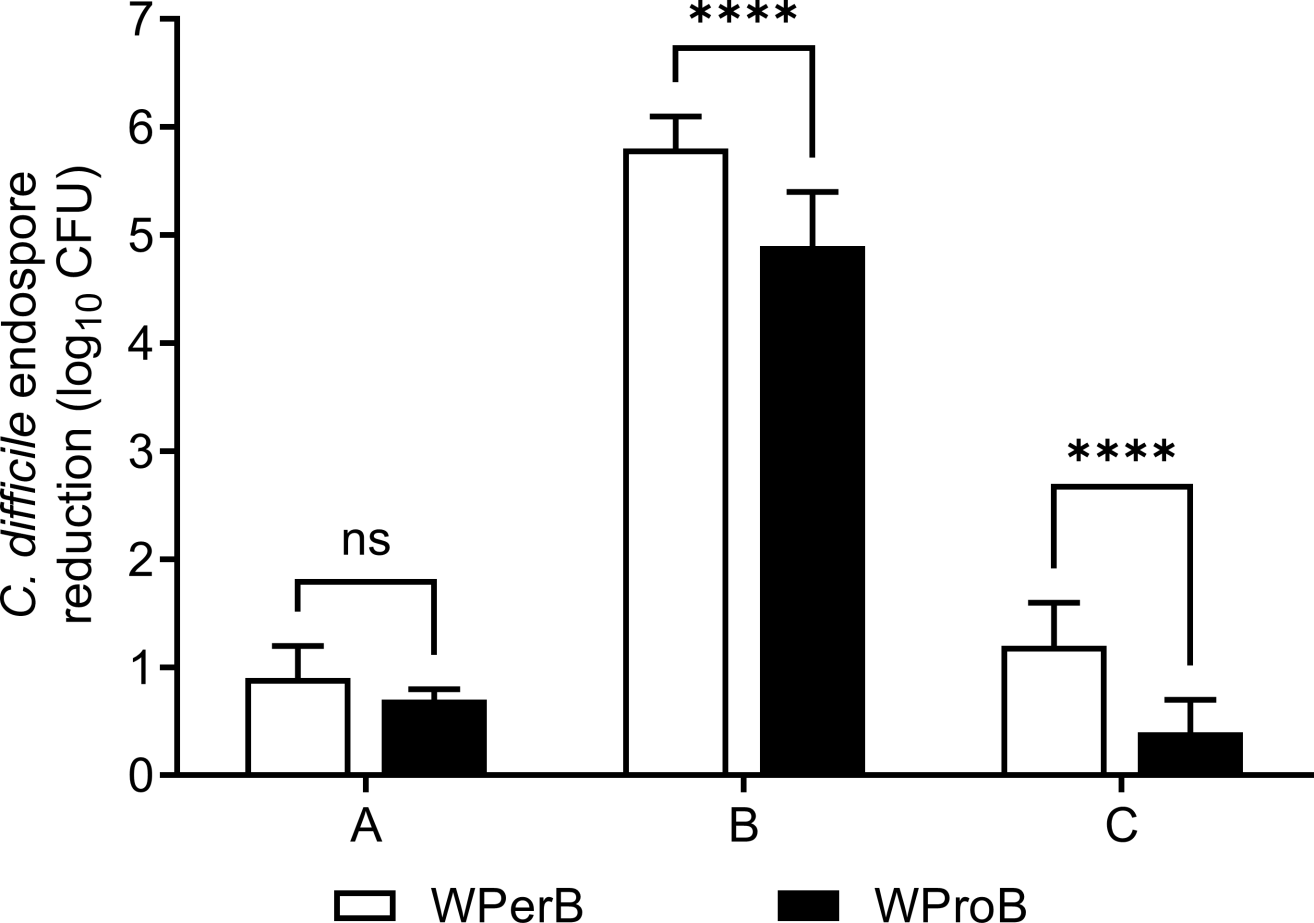
Efficacy of disinfectants against *C. difficile* endospores on carpet with different backings. Products A (0.5% H_2_O_2_), B (3.13% H_2_O_2_), and C (1,000 ppm sodium hypochlorite) were tested for an exposure time of 30 min. White bars indicate the efficacy of disinfectants on WPerB; black bars indicate the efficacy of disinfectants on WProB. Error bars represent standard deviations from 10 replicates in two independent experiments. The *P* values between two types of carpets for each disinfectant treatment were ≥0.05 (ns) and <0.0001 (****), respectively.

**Fig 3:**
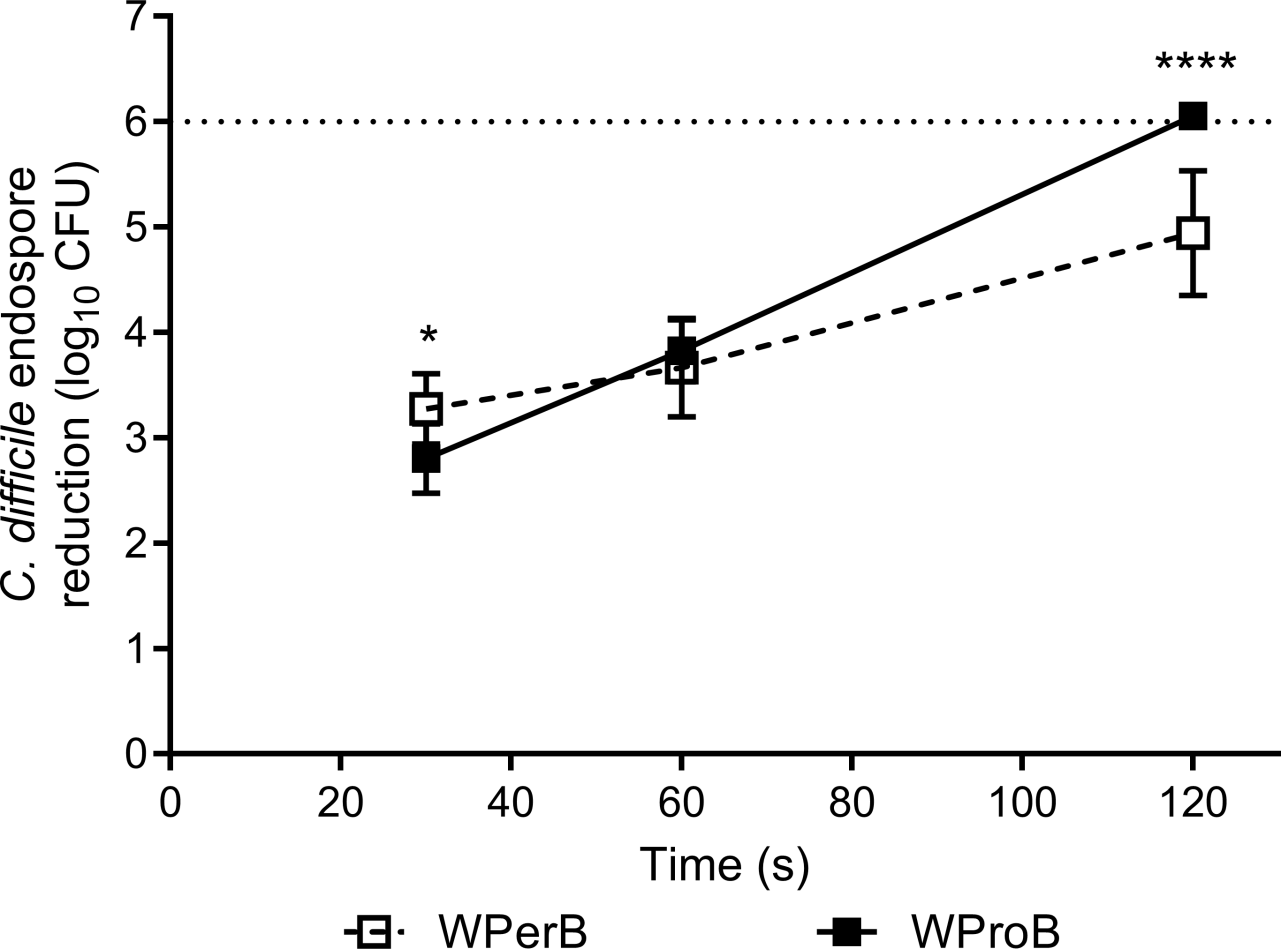
Efficacy of steam against *C. difficile* endospores on carpet with different backings. The dashed line with open squares represents efficacy on WPerB, and the solid line with solid squares represents efficacy on WProB. Error bars represent standard deviations from replicates in two independent experiments. The dotted line indicates the reach of the detection limit, which is equal to a 6 log_10_ CFU reduction. The *P* values among treatments at each experimental interval were <0.05 (*) and <0.0001 (****), respectively.

Hydrogen peroxide-based products A and B (Table 3) resulted in 0.9 and 5.8 log_10_ CFU reductions of *C. difficile* endospores on carpet with WPerB after a 30 min exposure time, respectively, as compared to a 0.7 and 4.9 log_10_ CFU reductions of *C. difficile* endospores on carpet with WProB, respectively (Fig. 2). Chlorine-based product C resulted in a higher reduction of *C. difficile* endospores on WPerB (1.2 log_10_ CFU reduction, compared to 0.4 log_10_ CFU on WProB). Steam resulted in 3.3, 3.7, and 4.9 log_10_ CFU reductions of *C. difficile* endospores on WPerB after 30, 60, and 120 s, respectively (Fig. 3). In contrast, the steam resulted in 2.8, 3.8, and >6.0 log_10_ CFU reductions of *C. difficile* endospores after 30, 60, and 120 s on WProB, respectively.

**TABLE 3:** Selection of EPA-registered disinfectants

Product	Active ingredient	Working concentration	Working pH	Neutralizer (concentration)
A	0.5% hydrogen peroxide	Ready-to-use	2.64	Catalase (1,300 U/mL)
B	3.13% hydrogen peroxide/0.099% octanoic acid/0.05% peracetic acid	Ready-to-use	2.95	Catalase (1,300 U/mL)
C	6% sodium hypochlorite	Diluted to 1,000 ppm	10.01	Fetal bovine serum (5%) + sodium thiosulfate (0.1%)

**Fig 4:**
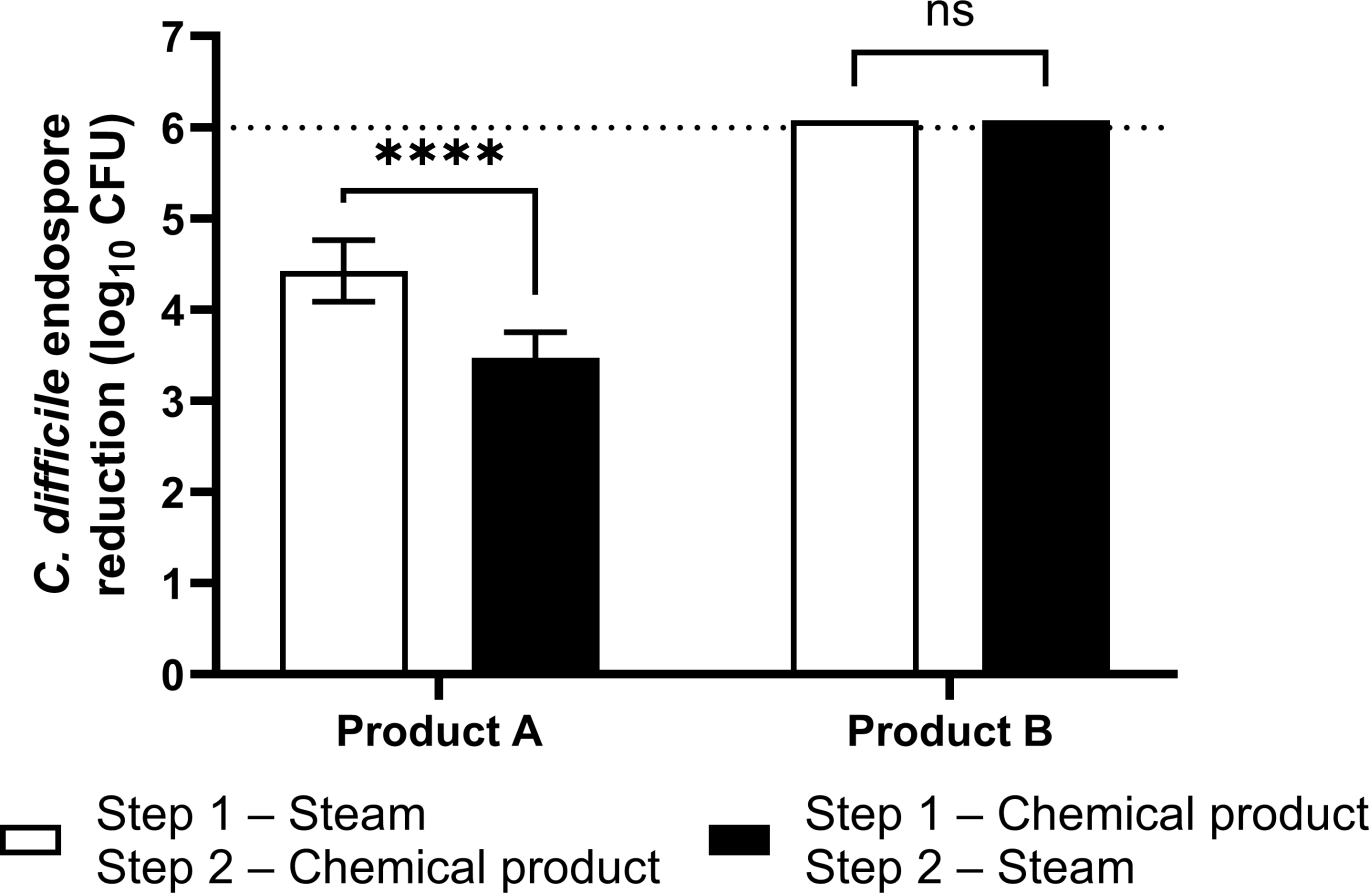
Efficacy of different application orders of steam and products A and B against *C. difficile* endospores on the WPerB carpet. In the first treatment sequence (white bars), steam was applied for 30 s, followed by a 30 min application of chemical products. In the alternative sequence (black bars), the chemical products were applied first for 30 min, followed by a 30 s steam treatment. The dotted line indicates the reach of the detection limit, which equals a 6 log_10_ CFU reduction. The *P* values among treatments at each application sequence were ≥0.05 (ns) and <0.0001 (****), respectively.

Figures 2 and 3 revealed that neither the three disinfectants nor steam alone could achieve a six log_10_ CFU reduction of *C. difficile* endospores on WPerB. To increase the overall disinfection efficacy of either steam or chemical disinfectants on WPerB, a combination of H_2_O_2_-based products A and B with steam was tested against *C. difficile* endospores. The combination of chlorine-based product C and steam was not tested, as it could potentially produce hazardous by-products. The combination of 30 s steam treatment and 30 min treatment using product B enhanced the overall efficacy on WPerB (Fig. 4), achieving >6.1 log_10_ CFU reduction in both application orders. On the other hand, the application orders for steam and product A significantly affected the overall efficacy. Specifically, a 30 s steam treatment followed by a 30 min application of product A reduced 4.4 log_10_ CFU of *C. difficile* endospores, while only a 3.5 log_10_ CFU reduction was observed in the reverse order.

### Effect of steam on carpet

**Fig 5:**
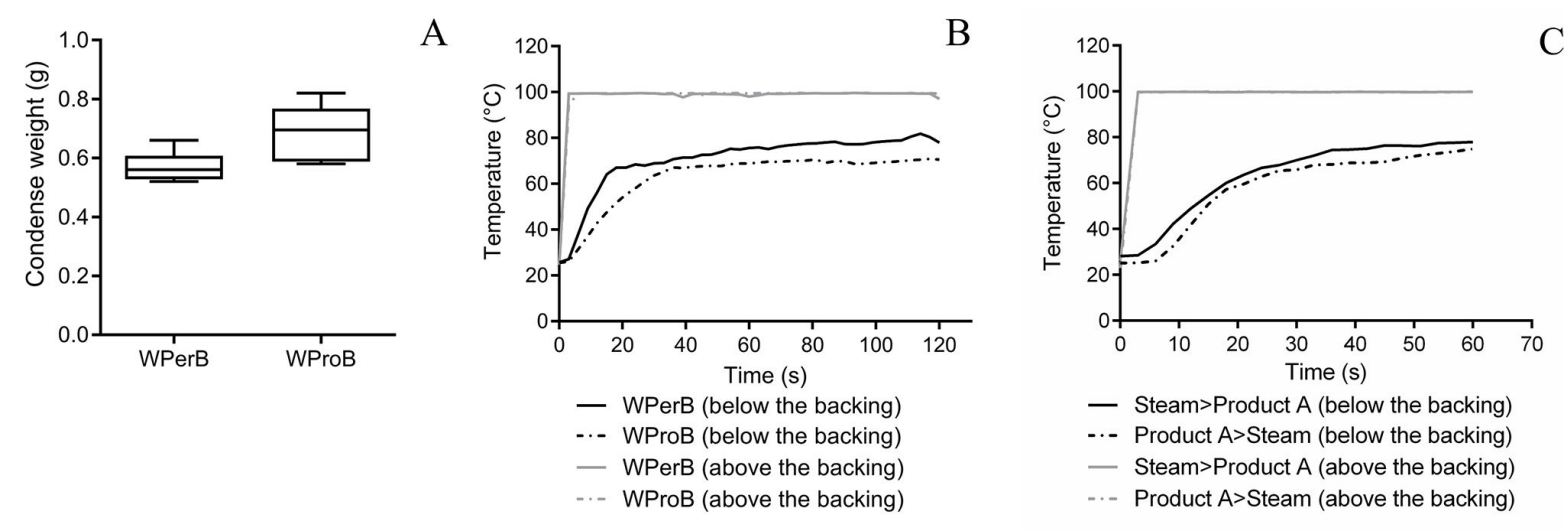
The condensed water weights of two types of carpets following steam treatment for 120 s (**A**), temperatures above and below the backings of two carpets over 120 s steaming (**B**), and temperatures above and below the backings of WPerB over 60 s steaming in combination of product A treatment (**C**). Each line indicates a mean temperature measurement from six replicates (B and C).

After a 120 s steam treatment, the treatment resulted in 0.57 g of condensed water on WPerB and 0.69 g on WProB (Fig. 5A). The temperature increased rapidly and stayed at around 99.3°C above both types of backing between 3 and 120 s (Fig. 5B). However, below the WPerB and WProB, the maximum temperatures reached were 81.83°C and 70.86°C, respectively. Clearly, the temperature below the WPerB was significantly higher than that for WProB during the 120 s exposure time (*P* = 0.0082), suggesting steam passed through WPerB. When the carpet was treated with steam before product A was applied, the maximum temperature reached was 77.95°C below WPerB during the 60 s exposure time (Fig. 5C). However, when the order of treatment was reversed, with product A applied before steam treatment, the maximum temperature achieved was reduced to 74.96°C.

## DISCUSSION

Testing disinfection efficacy on carpet is difficult due to the complex nature of carpet and the low recovery rate of target microorganisms. In this study, we first improved the microbial recovery method for *C. difficile* endospores on carpet. Subsequently, the disinfection study was conducted on both WPerB and WProB carpets. Among three EPA-registered chemical disinfectants, only product B was found efficacious against *C. difficile* endospores on carpet after 30 min. Additionally, steaming, as an alternative to chemical disinfectants, demonstrated effectiveness on carpet. To enhance disinfection efficacy, we explored combining chemical and physical approaches.

Disinfectants (e.g., H_2_O_2_ vapor, gaseous plasma, and UV-C radiation) have been tested against *C. difficile* endospores on non-porous surfaces, but conducting efficacy tests on porous surfaces is more challenging due to the lack of an effective recovery method (29–32). Sadowsky et al. (28) reported that the addition of a surfactant, such as Tween-80, in the buffer significantly enhanced the recovery of *C. difficile* endospores from 10.0% to 39.8% on olefin carpet. Our initial results showed that adding Tween-80 alone increased the recovery rate from 8% to 22% on nylon carpet. We then improved our sample preparation method using sonication and a stomacher to vigorously detach *C. difficile* endospores from the carpet samples. Our optimized recovery method (0.2% Tween-80, sonication, and stomaching) with over 60% recovery rate was more effective than the reported <40% recovery rate in the previous studies (28, 33).

Our results also revealed that the presence of soil decreased the recovery rate of *C. difficile* endospores from nylon carpet. This suggests that a large number of *C. difficile* endospores may persist on carpet following improper vacuuming, resulting in a high exposure risk of occupations to *C. difficile* endospores without a disinfection step. Additionally, the presence of soil significantly weakened the efficacy of surface disinfectants (34). This highlights the necessity of a thorough cleaning, such as vacuuming prior to the disinfection procedure to maximize the efficacy of disinfectants.

**Fig 6:**
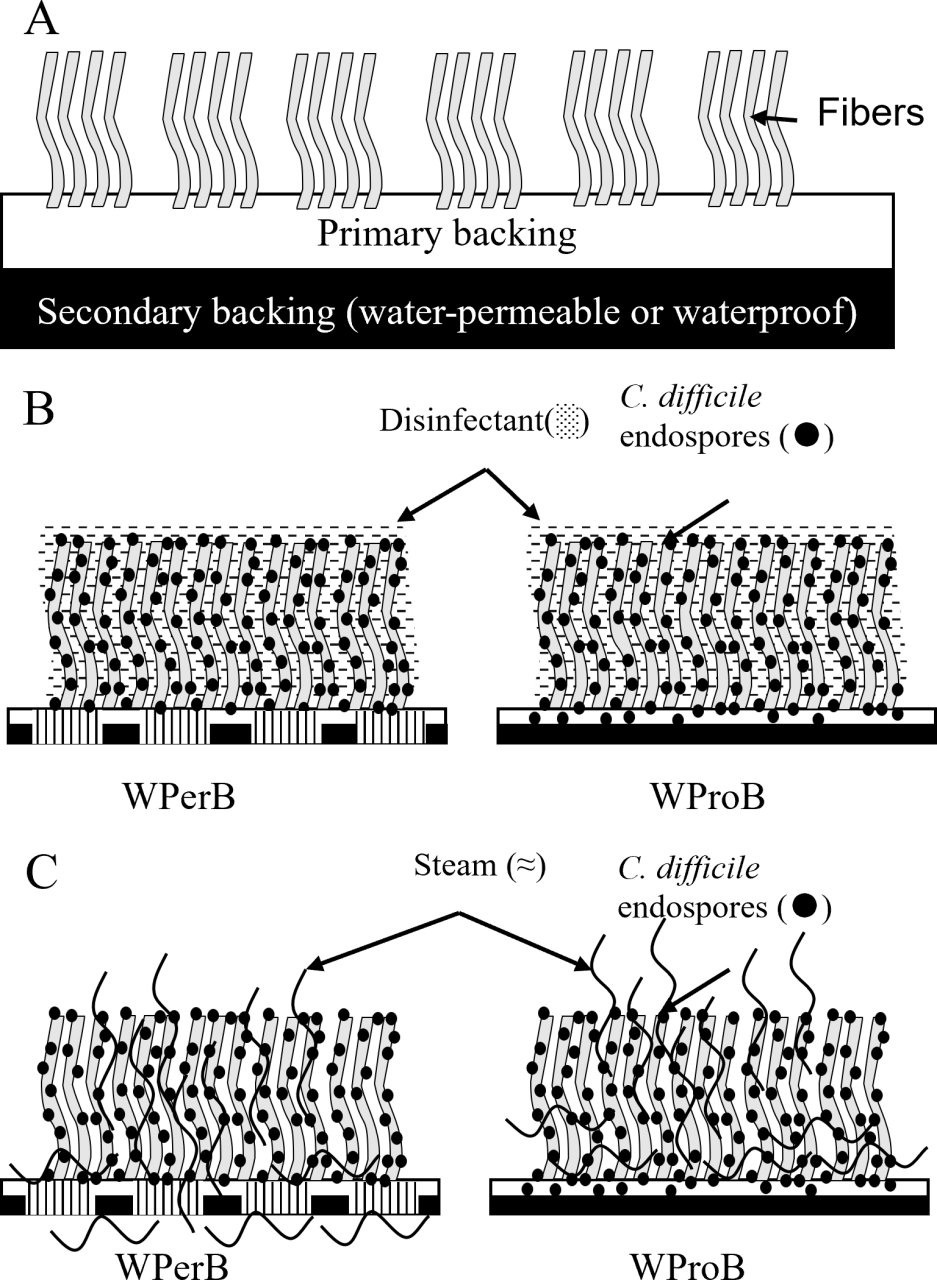
A general schematic of carpet with backings. WPerB and WProB are constructed with both primary and secondary backings (**A**). WPerB has a water-permeable backing (double layer with stripes), while WProB has a waterproof backing (double layer, **B**). Distribution of disinfectants and *C. difficile* endospores on two tested carpets (**B**). Distribution of steam and *C. difficile* endospores on two tested carpets (**C**).

*C. difficile* endospores were found to be more sensitive to chemical disinfectants on WPerB compared to WProB. This difference in sensitivity might be due to some differences in the distribution patterns of microorganisms and disinfectants in the carpet. The fibers in carpet backings tended to bind in clusters, which did not fully cover the entire backing (Fig. 6A and B). According to Carr et al. (35), a high content of water (50% dry weight of carpet) was retained in carpet fibers with backing that received less liquid due to capillary action. As a result, some of the primary backings might not receive chemical disinfectants during the application of disinfectants. On the other hand, a study revealed that blood drops penetrated fibers and even into the primary backing, which could suggest microorganism inoculum could also penetrate through fibers to backings due to the size of droplets other than the capillary action (35, 36). Moreover, the hydrophobic *C. difficile* endospores can attach and be absorbed by the primary layer of a more hydrophobic and waterproof backing used in WProB (Fig. 6B), which was confirmed by higher plate count of *C. difficile* endospores in the backing of WProB (Fig. 1). Thus, *C. difficile* endospores resided in the backing layer of WProB, making them barely accessible and shielded from the chemical action of disinfectants.

Steam effectively acted as a biocide due to its high temperature and penetration, which kills microorganisms (37). In addition, the heat transfer to suspensions or surfaces led to indirect inactivation of the microbes (38). For example, steam has been reported as efficacious against norovirus, molds, and antibiotic-resistant bacteria on carpet and other hard surfaces (18, 38–40). Moreover, some studies reported that *C. difficile* endospores can be inactivated by steam on stainless steel carriers or temperature at ≥75°C in suspension (41, 42). This is the first study reporting the efficacy of steam against *C. difficile* endospores on carpet. Different efficacies were observed on two types of carpet because of the interactions between steam and carpet backings. When the steam first contacted a dried carpet coupon, it quickly penetrated carpet fibers and heated the backing, while a fraction of the steam condensed on carpet backings and fibers (Fig. 6C). Based on the temperature monitoring results and condensed water measurements (Fig. 5), the steam quickly went through WPerB, while WProB could block the steam flow through the backing and retain more heat and condensed water within the carpet. As a result, *C. difficile* endospores are more inactivated (*P* < 0.05) on WProB than WPerB (Fig. 6C).

We also observed a mixed effect in the efficacy of steam against *C. difficile* endospores when used in conjunction with the application of product A on WPerB, compared to using either method alone. This effect likely stems from the distinct distribution patterns of steam and aqueous disinfectants on carpet (Fig. 6). Applying steam first could effectively reach most of the *C. difficile* endospores on the carpet in a short time, while product A or B, applied next, was able to stay in carpet fibers and most of backings for ensuring a prolonged exposure time. However, the reversed application order led to a reduction in the maximum temperature attainable on the carpet. This decrease in temperature was caused by the presence of product A, an aqueous disinfectant, on the carpet, which subsequently weakened the efficacy of the steam treatment. Therefore, steam treatment should be applied prior to chemical disinfectants to achieve a higher level of efficacy.

Despite our investigation into potential routine disinfection methods for disinfecting *C. difficile* endospores on carpet, limitations were encountered. First, EPA-registered disinfectants are intended for use on inanimate surfaces, such as hard non-porous surfaces, and soft porous surfaces (43, 44). However, there are limited commercial products for certain types of soft surfaces, such as upholstery and carpet (45). Thus, our results can only demonstrate potential disinfection strategies. Second, variations in sensitivity to disinfection were noted among different ribotypes of *C. difficile*, in addition to differences in virulence (28). Thus, other ribotypes of *C. difficile* endospores need to be examined. Third, even though we proposed a testing condition to simulate the real-world application, our data collected from lab conditions may differ from applications in real-world settings, such as long-term care facilities, which have more complicated environments (i.e., carpet specification, airflow, and disinfection frequency).

### Conclusion

We developed a modified microbial recovery protocol that can be used to accurately determine the efficacy of disinfectants against *C. difficile* endospores on carpet. A 120 s steam treatment was more effective than any of the three disinfectants tested. The efficacy of disinfectants is significantly influenced by the type of carpet backing, underscoring the need for informed guidance in selecting carpet materials in healthcare settings to enhance microbial safety. Additionally, when the selection of disinfectants in public settings is limited, such as product A, a disinfectant with known weaker efficacy, applying steam first enhanced the efficacy against *C. difficile* endospores, which may be a promising approach for disinfection of *C. difficile* endospores on carpet with water-permeable backings.

## MATERIALS AND METHODS

### Preparation and purification of *C. difficile* endospores

*C. difficile* ATCC 43593 (toxin A− and toxin B−) was cultured on modified brain heart infusion agar plates containing 5 g/L yeast extract, 1 g/L cysteine, and 1 g/L sodium taurocholate (BHIA/YE/CYS/T) and incubated inside an anaerobic chamber (BactronEZ; Sheldon Manufacturing, Oregon, USA) at 37°C for 7 days as previously described (15). Then, all plates were sealed with parafilm (Pechiney, Illinois, USA) and incubated at ambient conditions for another 7 days. Each agar plate was then flooded with 5 mL of 0.01 M phosphate-buffered saline (PBS) with 0.1% Tween-80, and the colony mass was scraped from the agar plates using sterile cotton swabs. The cell suspension was washed five times with ice-cold sterile deionized water, followed by centrifugation at 7,000 × *g* for 5 min at 4°C. Vegetative cells of *C. difficile* were removed by gradient centrifugation in 50% (w/vol) sucrose solution (46), and the endospore suspension was washed three times with sterile ice-cold water. The concentration of endospores was enumerated on BHIA/YE/CYS/T plates, and the purity of prepared endospores was confirmed under a microscope after endospore staining. The stock culture of *C. difficile* endospores at ca. 10^8^ CFU/mL was stored at 4°C for routine tests and at −80°C for long-term storage. Before the recovery optimization and efficacy test, *C. difficile* endospores were prepared with the soil load by mixing 340 µL of endospore suspension with 25 µL of 5% bovine serum albumin, 35 µL of 5% yeast extract, and 100 µL of 0.4% bovine mucin according to ASTM E2197-17 (47).

### Selection of disinfectants and preparation of carpet coupons

One chlorine-based and two H_2_O_2_-based disinfectants (Table 3) were selected based on the effectiveness against *C. difficile* endospores from a previous study (15), although all three products do not have a disinfection claim against *C. difficile* endospores on carpet. To test the efficacy of steam cleaners, a household steam cleaner (IVASTEAMR20; Ivation, New Jersey, USA) was chosen for this study. Specifically, the steam cleaner had a 1.8 L water tank and could reach a maximum temperature of 170°C and a maximum pressure of 500 kPa. The nylon loop pile carpet Color Accent with WPerB (Shaw Inc., Georgia, USA) was selected according to the Carpet and Rug Institute Test Method 112 (48). To compare the effect of carpet backing, another nylon loop pile carpet, Highlight with WProB (Shaw Inc.), was chosen to have almost the exact specifications of color accent except for the backing (Table 4). Although both carpets contained antisoil coatings, the carpets were confirmed as free of sporicidal activities. The carpets were cut into 5 × 5 cm^2^ coupons with a mechanical cutting die (Model 1500; Freeman Schwabe, Ohio, USA) (kindly provided by Dr. Daniel Price; Interface Inc., Georgia, USA) then dusted by hand to remove loose fibers. Before the tests, the carpet coupons wrapped in aluminum foil were autoclaved on a 30 min dry cycle and cooled at room temperature overnight.

**TABLE 4:** Characteristics of tested carpet

Specifications	WPerB	WProB
Commercial name	Color accent	Highlight
Fiber (construction method)	Nylon 6 (loop)	Nylon 6 (loop)
Fiber finishing	Solution-dyed, soil-resistant coating	Solution-dyed, soil-resistant coating
Average density (g/cm^3^)	0.348	0.317
Finished pile thickness (mm)	2.92	3.20
Primary backing	Synthetic	Synthetic
Secondary backing	Stalok	Ecoworx Performance Broadloom
Backing to moisture	Water permeable	Waterproof
Backing thickness (mm)	3.96	3.56

### Recovery optimization of *C. difficile* endospores from nylon carpet

As the addition of Tween-80, a common surfactant, can improve the recovery rate of *C. difficile* endospores from carpet (28), the concentration of Tween-80 in the neutralizer was optimized. Each of the six pre-cut WPerB carpet coupons received 100 µL *C*. *difficile* spore suspension (~10^8^ CFU/mL) spiked into 10 spots with or without soil load and then dried for 1 h under ambient conditions inside a biosafety cabinet; relative humidity was maintained at 40% ± 10% in the lab and confirmed by a humidity meter (VWR, Pennsylvania, USA). After drying, three coupons were immediately transferred into a flask with 100 mL of PBS plus 0.02%, 0.1%, and 0.2% Tween-80, respectively. All flasks were ultrasonicated for 1 min at 40 kHz (FS110; Fisher Scientific, Pennsylvania, USA) and then vigorously shaken at 250 rpm in a shaking incubator (New Brunswick Scientific C25, New Jersey, USA) for 15 min, followed by *C. difficile* endospore enumeration on BHIA/YECYS/T plates.

Our preliminary study showed that the combination of sonication and shaking could not effectively recover *C. difficile* endospores from the carpet. Therefore, a stomacher (Seward, New York, USA) was employed to improve the recovery rate of *C. difficile* endospores by optimizing the stomaching time. Each of the six pre-cut WPerB carpet coupons received 100 µL *C*. *difficile* spore suspension and dried as described above, and the dried coupons were immediately transferred into a flask with 100 mL of PBS plus 0.2% Tween-80. All flasks were ultrasonicated for 1 min at 40 kHz and then vigorously stomached at 200 rpm in a stomacher for 1, 2, and 3 min. *C. difficile* endospore population from the inoculum suspension and all carpet coupons were enumerated on BHIA/YE/CYS/T plates. The optimal recovery method was eventually verified on WProB carpet during the efficacy testing.

### Determination of *C. difficile* endospore distribution on carpet

*C. difficile* endospores were inoculated and dried on carpet as described above. Then the fibers of the carpets were removed by using sterile disposable scalpels, while *C. difficile* endospores on control coupons were directly recovered. The endospores on the two different types of carpet backing were recovered as described above.

### Efficacy test on carpet

As no standard efficacy testing method was available for carpet, the efficacy of chemical disinfectants and steam against *C. difficile* endospores on carpet was evaluated as previously described with some modifications (18). In brief, each pre-cut coupon received 100 µL of *C. difficile* endospore suspension, spiked into 10 spots and then dried for 1 h under ambient conditions with relative humidity being monitored and controlled at 40% ± 10%. After drying, unscrubbed control coupons (*n* = 3) were immediately transferred into a flask with 100 mL of PBS plus 0.2% Tween-80, and spores were directly recovered to verify the recovery efficiency. Each treatment coupon (*n* = 5) received about 6 mL of disinfectants, which was sprayed for ca. 8 s on each carpet coupon at a distance of ca. 12 cm using Preval sprayers (Nakoma, Illinois, USA). Then coupons were scrubbed clockwise and counterclockwise for 30 s each with a disinfectant-saturated (approximately 1 mL) surgical scrub brush (E-Z Scrub, Becton Dickinson, New Jersey, USA) and left at ambient conditions for an exposure time of 30 min. For scrubbed control (n=3), the disinfectant was replaced by PBS. After the 30 min exposure time, scrubbed control coupons and testing coupons were transferred into a flask with 100 mL of appropriate neutralizers plus 0.2% Tween-80 (Table 3).

As for the steam treatment, the head of the steam cleaner was wrapped with sterile terry cloth and folded into four layers. The head was saturated by the steam for 10 s before each application, and then coupons were scrubbed vertically for an exposure time of 30, 60, and 120 s with steam, respectively. For the scrubbed controls, coupons were scrubbed by the wrapped head of the steam cleaner without heat for 120 s. Scrubbed control coupons (n=3) and treatment coupons (*n* = 5) were immediately transferred into a flask with 100 mL of PBS plus 0.2% Tween-80. All flasks were ultrasonicated for 1 min at 40 kHz and then vigorously stomached at 200 rpm for 3 min. The *C. difficile* endospore population in the inoculum suspension and on all carpet coupons was enumerated on BHIA/YECYS/T plates.

To increase the disinfectant efficacy, thus, a combination approach, i.e., applying product A or B for 30 min then followed by treatment with steam for 30 s and the reverse application order, was explored on the carpet with WPerB as described above. Three replicates of treatment coupons were used for each trial.

### Measurements of carpet treated by the steam cleaner

During steam treatment of the carpet, condensed water accumulated underneath. The condensed water was measured by weighing carpet coupons before and after 120 s of steaming. Meanwhile, temperatures above and below the carpet backing were immediately measured in triplicate by type T thermocouples (HotMux; DCC Corporation, New Jersey, USA) and recorded every 3 s. All the above experiments were repeated once.

### Statistical analysis

Ten replicates of each product were tested in two independent efficacy tests on the carpet. Reductions were calculated by log_10_ (*N_0_*/*N_d_*), where *N_d_* is the average *C. difficile* endospore population from the treatment coupons and *N*_*0*_ is the average *C. difficile* endospore population from the scrubbed control coupons. Statistical analysis was performed using a one-way multiple-comparison analysis of variance to determine the relationship between disinfectants and reduction. All results were expressed as mean ± standard deviation. Statistical significance was defined as a *P* value of <0.05 to establish a more conservative estimate of efficacy. Statistical analyses were conducted using GraphPad Prism version 6.01 (GraphPad Software, Inc., California, USA).
